# Exploring the anti-inflammatory potential of topical hyaluronic acid for vocal fold injury in a rat model

**DOI:** 10.1007/s00405-023-08278-1

**Published:** 2023-10-12

**Authors:** David Hortobagyi, Tanja Grossmann, Andrijana Kirsch, Christina Winter, Eva Roblegg, Markus Gugatschka

**Affiliations:** 1 Division of Phoniatrics, ENT University Hospital, Medical University of Graz, Auenbruggerplatz 26, 8036 Graz, Austria; 2 Institute of Pharmaceutical Sciences, Pharmaceutical Technology and Biopharmacy, University of Graz, Graz, Austria

**Keywords:** Vocal fold inflammation, Vocal fold injury, Hyaluronic acid, Diclofenac, Topical treatment

## Abstract

**Purpose:**

Vocal fold injuries are associated with fibrosis and dysphonia, which is a major obstacle to surgical treatment. The aim of this study is to evaluate the effect of topical hyaluronic acid with or without diclofenac on the inflammatory phase of vocal fold wound healing.

**Methods:**

Forty-one male Sprague–Dawley rats were randomly assigned to four groups: an uninjured control group, an injured control group without any treatment, and two intervention groups in which hyaluronic acid with or without diclofenac was applied to the injured vocal fold. Gene expression of inflammatory markers and ECM-related molecules were examined.

**Results:**

Vocal fold injury resulted in a significant upregulation of inflammatory parameters [*Ptgs2*, *Il1b* and *Il10*] and *Has1*. *Tgfb1*, *Has3* and *Eln* gene expression were significantly downregulated by the topical application of hyaluronic acid. The combination of hyaluronic acid and diclofenac did not result in any significant changes.

**Conclusions:**

Vocal fold wound healing was significantly improved by a single post-operative topical application of hyaluronic acid. The addition of diclofenac may provide no additional benefit.

**Supplementary Information:**

The online version contains supplementary material available at 10.1007/s00405-023-08278-1.

## Introduction

Phonation is a complex process that is caused by vocal fold (VF) vibrations. The integrity of these delicate anatomical structures is essential for voice production, speech intelligibility, and ultimately quality of life [1]. Following VF injury wound healing processes are initiated to restore organ integrity and barrier function. Welham et al. showed already in 2008 that multiple mRNA levels of inflammatory (*Ptgs-2, Tnfa, Il1b*) and ECM encoding genes (*Has-1*) were upregulated following 1 h after injury [2]. This first phase of wound healing, the inflammatory phase, is particularly important as it paves the way for the subsequent steps. Even small deviations in this phase can lead to a disruption of the transition to the second (proliferative) phase of wound healing and eventually to scarring. In case of the VF, this leads to deterioration of the vibrational properties and permanent dysphonia [3].

Given the importance of intact VFs, a number of strategies have been developed to overcome these problems after injury, such as the injection of various types of stem cells [4]. Another approach is to inject active agents into the freshly injured mucosa to prevent damage to the VF lamina propria; hyaluronic acid (HA) is one such candidate.

This glycosaminoglycan is a major component of the extracellular matrix (ECM) and is known for its ability to store large amounts of water, making it essential for VF pliability. Animal studies have shown that the concentration of HA in the VF decreases after acute injury, leading to functional deficits [5]. In addition to its mechanical properties that improve pliability, HA also has bioactive effects that influence cell migration and proliferation, which has a direct impact on wound healing [6–11]. Injection of synthetic HA hydrogels has shown promising results in animal models [7, 9]. Currently, there is no hydrogel that perfectly mimics the complex composition of the ECM, making a replacement of injured tissue impossible.

Although HA is generally considered a safe substance, injection into the VF carries the risk of significant side effects, such as inflammation or edema [12]. An alternative that avoids the problems associated with injections is superficial application. This is a common method in other areas of medicine and is used to prevent scarring and to treat keloids [13, 14].

Diclofenac is a commonly used, non-steroidal anti-inflammatory drug. The rationale for its topical use is its ability to inhibit the activity of the enzyme cyclooxygenase (COX), which plays a crucial role in the production of prostaglandin, prostacyclin and thromboxane. By inhibiting COX, diclofenac is thought to have anti-inflammatory effects that promote wound healing. However, there are significant side effects. While topical application of diclofenac has a lower risk of systemic side effects, local skin irritation may have undesirable consequences for patients [15].

Currently, there is no substance used in clinical routine that can be applied topically after VF injury to prevent the consequences of impaired wound healing, such as fibrosis.

The aim of this study was to investigate the effects of HA-containing formulas with and without diclofenac on the early phase of wound healing when applied directly to the injured mucosa postoperatively in an in vivo rat model.

## Materials and methods

### Experimental setup

Forty-two rats were initially enrolled in the study and divided into four groups: an uninjured control group, an injured control group and two injured treatment groups in which the rats were treated with either topical HA alone or in combination with diclofenac. One rat was later excluded from the study as described below.

**Fig. 1:**
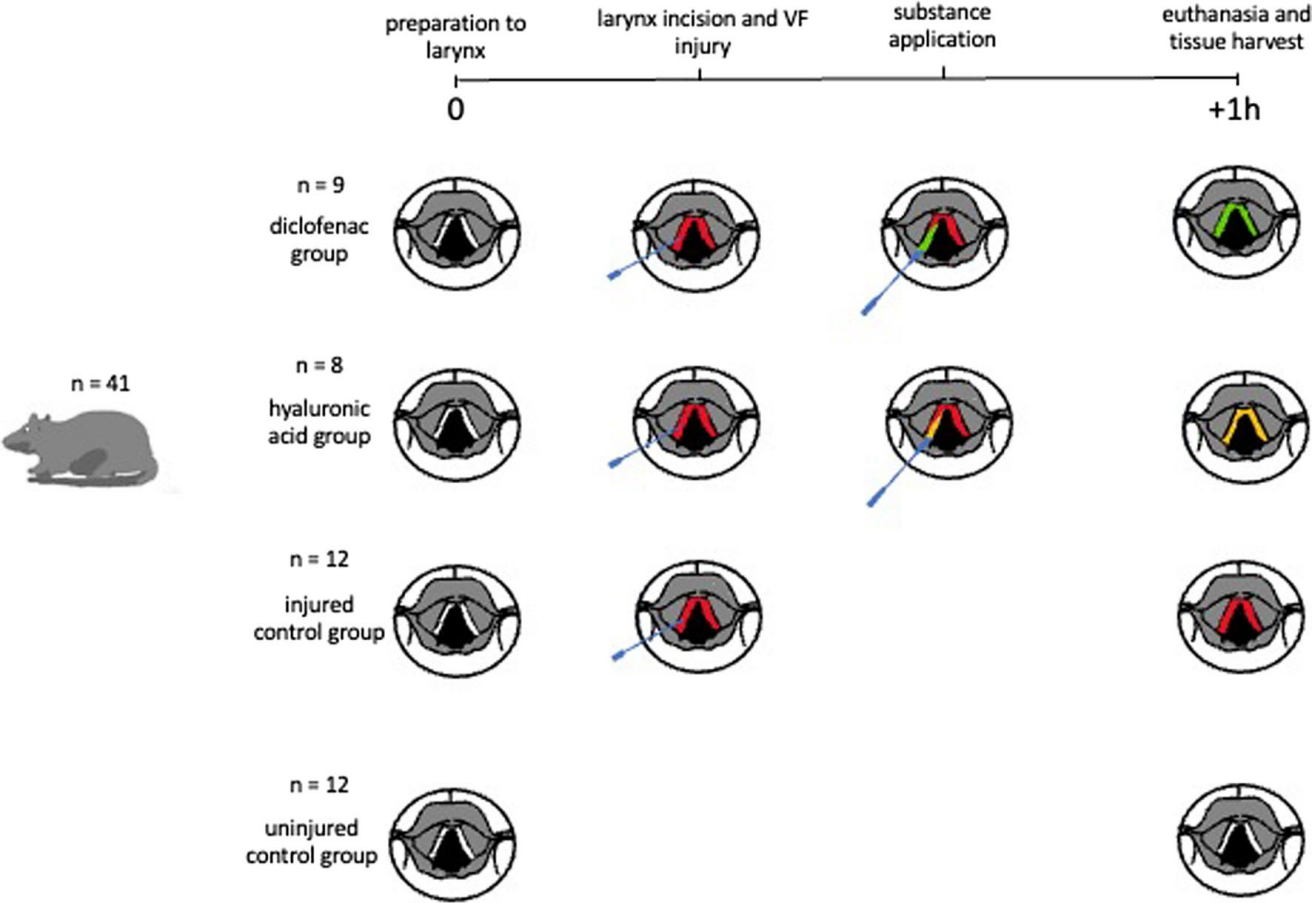
Experimental design. Forty-one adult male Sprague–Dawley rats were randomly assigned to four groups: an uninjured control group (white), an injured control group (red), and two treatment groups in which HA alone (yellow) or HA combined with diclofenac (green) was applied to the injured area. One hour after injury, rats were euthanized and VF mucosa was harvested and stored in cryotubes at – 80 °C until further processing

After induction of anesthesia, all rats had their necks shaved and dissected to expose the trachea. All rats, regardless of which group they were assigned to, were kept alive under a heat lamp for 1 h after the intervention. In the uninjured control group, surgery was stopped at this point. In the remaining animals, which were assigned to the injured groups, the trachea was opened and a cannula was inserted into the trachea. Under microscopic guidance, the larynx was split medially and after identification of the VF, these were injured bilaterally with a needle. The rats assigned to the injured control group were also placed under the heat lamp for 1 h. The remaining rats received topical therapy. Each of these steps is explained in more detail in the following sections and visualized in Fig. 1.

### Gel fabrication

2% (w/w) HA acid sodium salt (Mw 30.000–50.000 Da, Prod.No. 53747; Sigma Aldrich, Munich, Germany) was stirred in MilliQ water at 55 °C for 2–3 h in a closed vial until a clear gel was formed. Subsequently, 1.5% (w/w) diclofenac sodium was slowly added and the mixture was stirred for 15 min.

### Rheological investigations

The viscoelastic behavior in terms of storage modulus *G*′, loss modulus *G*″, and complex viscosity η* was investigated using a Physica MCR 301 rheometer (Anton Paar, Graz, Austria) and a CP-50-1 measurement system with cone-plate geometry at 37 °C. Shear rates between 0.1 and 10 rad/s were applied. The loss factor tan*δ* was calculated as the ratio of *G*″/*G*′. The thixotropy behavior of the gels was investigated using the same device in rotational measurement mode. The reduction and recovery of the structural strength of the gels was investigated by applying a constant shear stress of 250 rad/s for 60 s, followed by a rest period of 300 s.

### Slip angle and drop weight

The slip angles were evaluated using a glass microscope slide, and the drop weight of the gels was evaluated using the standard pharmacopeia method for liquid and semisolid formulations (Ph. Eur. 2.1.1).

### Animals

Forty-one male Sprague–Dawley rats (older than 12 weeks) with an average weight of 569.31 g ± 32.05 g were used. Rats were housed in pairs in the pathogen-free facility of the Medical University of Graz for 4–6 weeks for acclimatization with ad libitum water and standard food. All procedures were approved by the Austrian Federal Ministry of Science, Research and Economy (approval number: BMBWF-66.010/0083-V/3b/2018) and complied with the institution´s animal care guidelines.

For the experiments, rats were randomly assigned to four groups: an uninjured control group, an injured control group, and two treatment groups in which HA alone or HA combined with diclofenac was applied to the injured area (Fig. 1). One rat from the HA group was excluded due to lack of genetic material in the harvested tissue.

### Surgical procedure

**Fig. 2:**
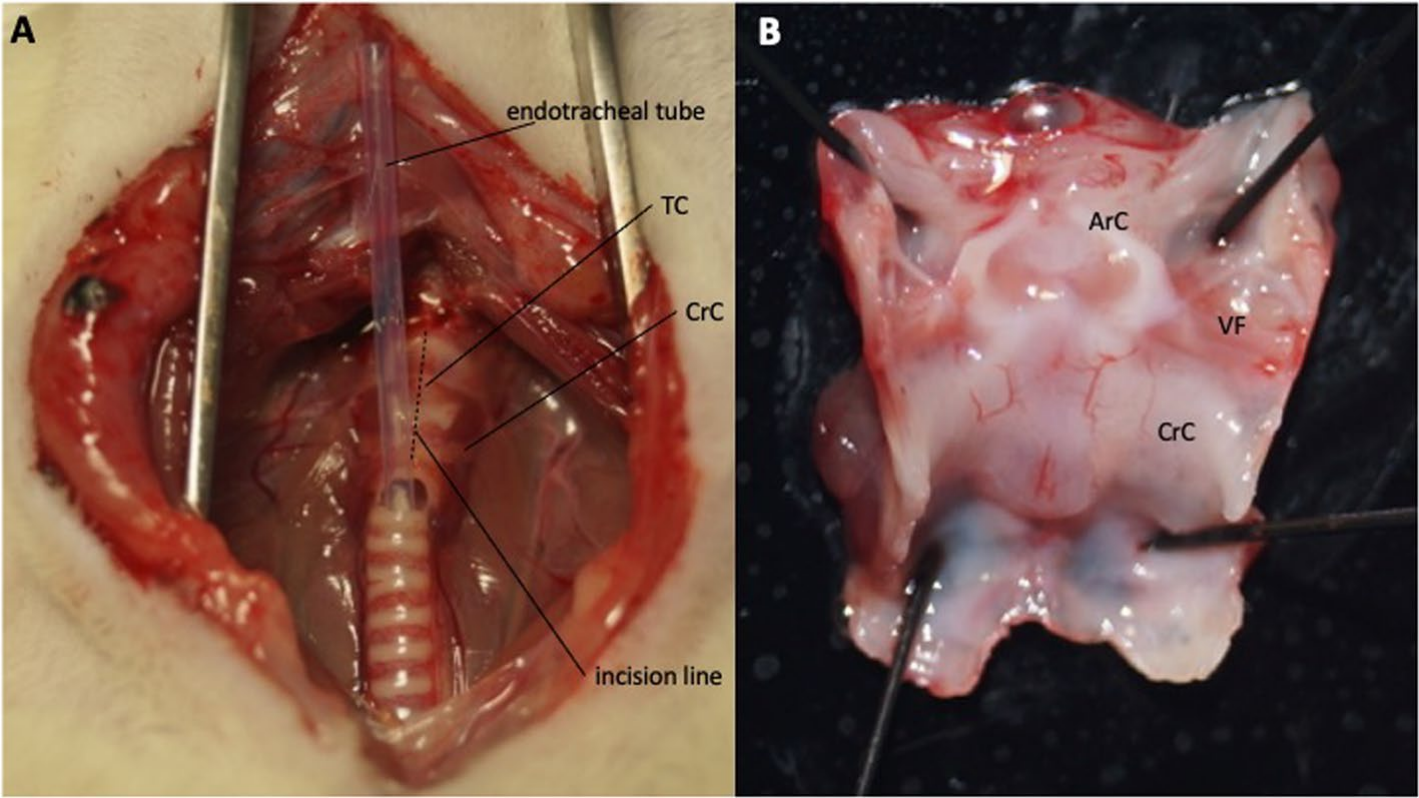
Representative images of the larynx. Intraoperative (**a**) and post excisional (**b**) view on the larynx. *TC* thyroid cartilage, *CrC* cricoid cartilage, *ArC* arytenoid cartilage, *VF* vocal fold

Anesthesia was induced by exposing the rats to isoflurane (5% delivered at 2 L/min) in an induction chamber for 10 min, followed by maintenance with intramuscular application of medetomidine (75–101.25 µg/kg), midazolam (1–1.35 mg/kg), and fentanyl (2.5–3.38 µg/kg). The neck was shaved anteriorly and disinfected with ethanol. Rats were then placed supine on an operating platform and a vertical midline incision was made with a No. 21 surgical blade. The pre-laryngeal tissue was bluntly retracted to avoid major injury and bleeding. In rats assigned to the uninjured control group, only the thyroid cartilage was visualized, but the larynx and trachea were not dissected. Rats assigned to VF injury were tracheostomized between the first and second tracheal rings. A plastic tube from a 17-gauge venous cannula was then inserted into the trachea for intubation (Fig. 2a). The larynx was dissected using a vertical median incision. VF injury was induced under the microscope (Vision SX45) by multiple vertical scratches with a 23-gauge needle. Minor bleeding due to mucosal injury was controlled by local compression with cotton swabs. Depending on the assigned group, either diclofenac or HA gel was applied to the injured VF. Rats in the injured control group were not exposed to any substance. The surgeon was blinded to the gel treatment applied.

After injury, animals were placed under a warming lamp to prevent hypothermia until tissue harvest.

### Tissue harvest

One hour after surgery, rats were euthanized by intracardiac injection of 1 mL pentobarbital sodium (400 mg/mL). The larynx was excised, mounted on a mounting board, and placed under a microscope (Fig. 2b). Mucus, blood, and gel were removed with cotton swabs, and the VF mucosa was harvested, snap frozen in liquid nitrogen in MagNa Lyser Green Beads tubes (Roche Diagnostics, Mannheim, Germany) and stored at -80 °C until further processing.

### RNA extraction and reverse transcription quantitative polymerase chain reaction (qPCR)

For tissue homogenization, 700 µl of QIAzol reagent (Qiagen, Hilden, Germany) was added to the frozen samples. Four homogenization steps (20 s at 6500 rpm) were performed on the MagNa Lyser instrument with intermittent cooling of the samples on ice for 1 min. Total RNA was then isolated using the miRNeasy Mini kit (Qiagen) according to the manufacturer´s instructions and RNA concentration was determined using the NanoDrop 2000c UV–Vis spectrophotometer (Thermo Scientific, Waltham, MA, USA). Reverse transcription (RT) was performed using the QuantiTect Reverse Transcription kit (Qiagen) according to the manufacturer´s protocol. RT-qPCR was performed as previously described [16]. All primers used are listed in Table 1. Samples were measured in technical triplicates and data were corrected using a Universal Rat Reference RNA (cat.no.QS0641; Invitrogen) for interplate calibration. Relative quantification of mRNAs of interest was calculated based on the 2-ΔΔCq method [17] with minor modifications: the geometric mean of the internal reference genes Werner helicase interacting protein 1 (*Wrnip1*) and ubiquitously expressed, prefoldin-like chaperone (*Uxt*) was used for internal normalization, and the mean of all analyzed samples was used as the reference to calculate ΔΔCq values.

**Table 1:** Primers for gene analysis with RT-qPCR

Gene	Gene symbol	Forward primer	Reverse primer
*Collagen type I alpha 1 chain*	*Col1a1*	GTACATCAGCCCAAACCCCA	TCGCTTCCATACTCGAACTGG
*Collagen type I alpha 2 chain*	*Col1a2*	TGTCGATGGCTGCTCCAAAA	CCGATGTCCAGAGGTGCAAT
*Collagen type III alpha 1 chain*	*Col3a1*	AGGATGGCTGCACTAAACACA	ATGATGGGGAGTCTCATGGC
*Cyclooxygenase 1*	*Ptgs1*	TTAGGCCATGGGGTAGACCTT	GGACGCCTGTTCTACGGAAG
*Cyclooxygenase 2*	*Ptgs2*	CCAACCCATGTCAAAACCGTG	TCTTGTCAGAAACTCAGGCGTAG
*Elastin*	*Eln*	Biorad, qRnoCED0001859	
*Hyaluronan synthase 1*	*Has1*	Biorad, qRnoCID0007192	
*Hyaluronan synthase 2*	*Has2*	CGTGGATTATGTACAGGTGTGTG	CTCCAACCATCGGGTCTTCT
*Hyaluronan synthase 3*	*Has3*	Biorad, qRnoCID0002186	
*Interferon gamma*	*Ifng*	Biorad, qRnoCID0006848	
*Interleukin 1 beta*	*Il1b*	AAATGCCTCGTGCTGTCTGA	AGGCCACAGGGATTTTGTCG
*Interleukin 10*	*Il10*	CCTTACTGCAGGACTTTAAGGGTT	CTGGGGCATCACTTCTACCAG
*Nuclear factor kappa B subunit 1*	*Nfkb1*	GGACAACTATGAGGTCTCTGGG	GCCTCTGTGTAGCCCATCTG
*Tumor necrosis factor*	*Tnf*	ATGGGCTCCCTCTCATCAGT	GCTTGGTGGTTTGCTACGAC
*Ubiquitously expressed, prefoldin-like chaperone*	*Uxt*	TTGAGCGACTCCAGGAAACTAA	CTGGGACCATTGTGTCAACG
*Werner helicase interacting protein 1*	*Wrnip1*	TGGTAAGGTTTGCCAGCGAG	GGCCAGAAGCACCTCACACT

### Statistical analysis

Statistical analysis was performed with SPSS (version 27). Normal distribution was assessed using the Shapiro–Wilk test. Significant differences were calculated using the Kruskal–Wallis test. For *p* values < 0.05, a post-hoc Dunn’s test was performed with Benjamini–Hochberg correction for multiple comparisons.

## Results

### Viscoelastic properties of the gel

**Fig. 3:**
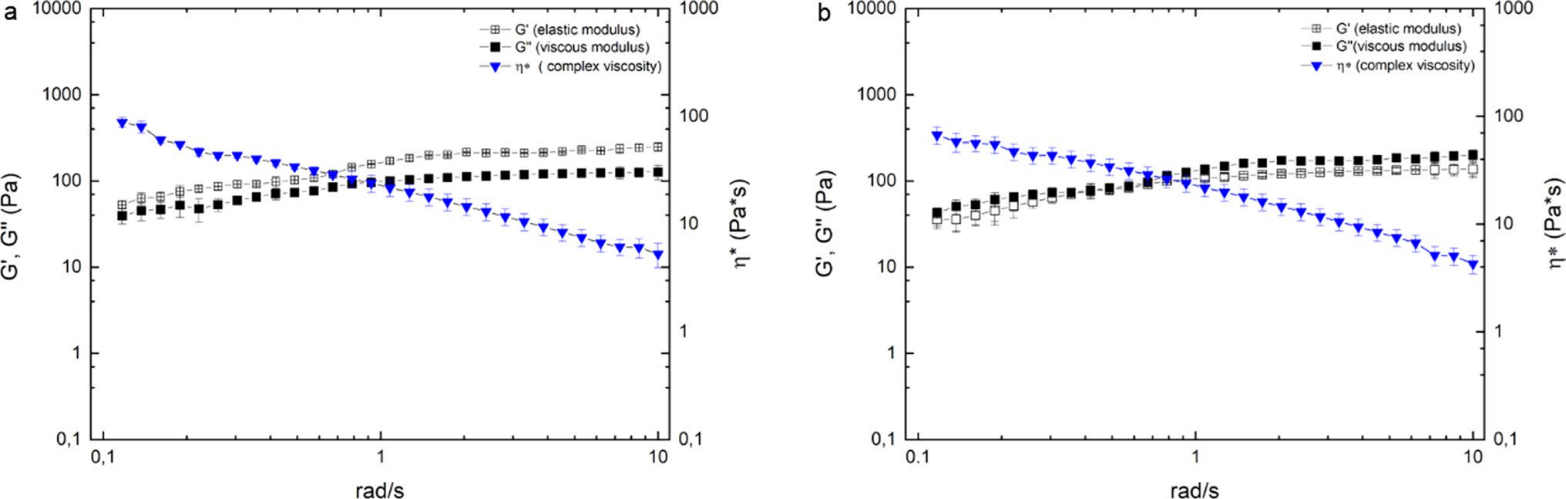
Viscoelastic properties of the gel. Viscoelastic properties of the 2% HA gel (**a**) and the 2% HA and 1.5% Diclofenac–Na gel (**b**) measured between 0.1 and 10 rad/s at 37 °C

The pure and drug loaded HA gels show a shear thinning and viscoelastic behavior. The viscosity of both gels is in a similar range. However, the viscoelastic properties differ slightly. For HA gels, the elastic modulus *G*′ dominates the viscous modulus *G*′′ over the applied shear range, resulting in tan*δ* values between 0.78 and 0.63. Through the addition of diclofenac–sodium, the viscous modulus is high at the starting point at 0.1 rad/s. After a crossover point at low shear rates, i.e., 0.6–0.8 rad/s, the elastic modulus dominates the viscous modulus indicating more stable network interactions. The loss factor tanδ increased from a starting value of 1.24 at 0.1 rad/s to 0.86 after 0.8 rad/s. Both gels exhibit a thixotropic behavior with a complete recovery of the structure as soon as the stress is stopped. This is particularly important for gel application with syringes, as the formulation liquefies due to shear stress, but rebuilds its structure within a very short time and thus remains at the site of action. The slip angle of the gels was 48° ± 2° for HA gels and 52° ± 1° for the drug-loaded gels, and the average drop weight (*n* = 20) was 8.044 ± 0.683 mg, which equals an average calculated drug loading of 0.12 mg/drop (Fig. 3).

### Inflammatory markers

**Fig. 4:**
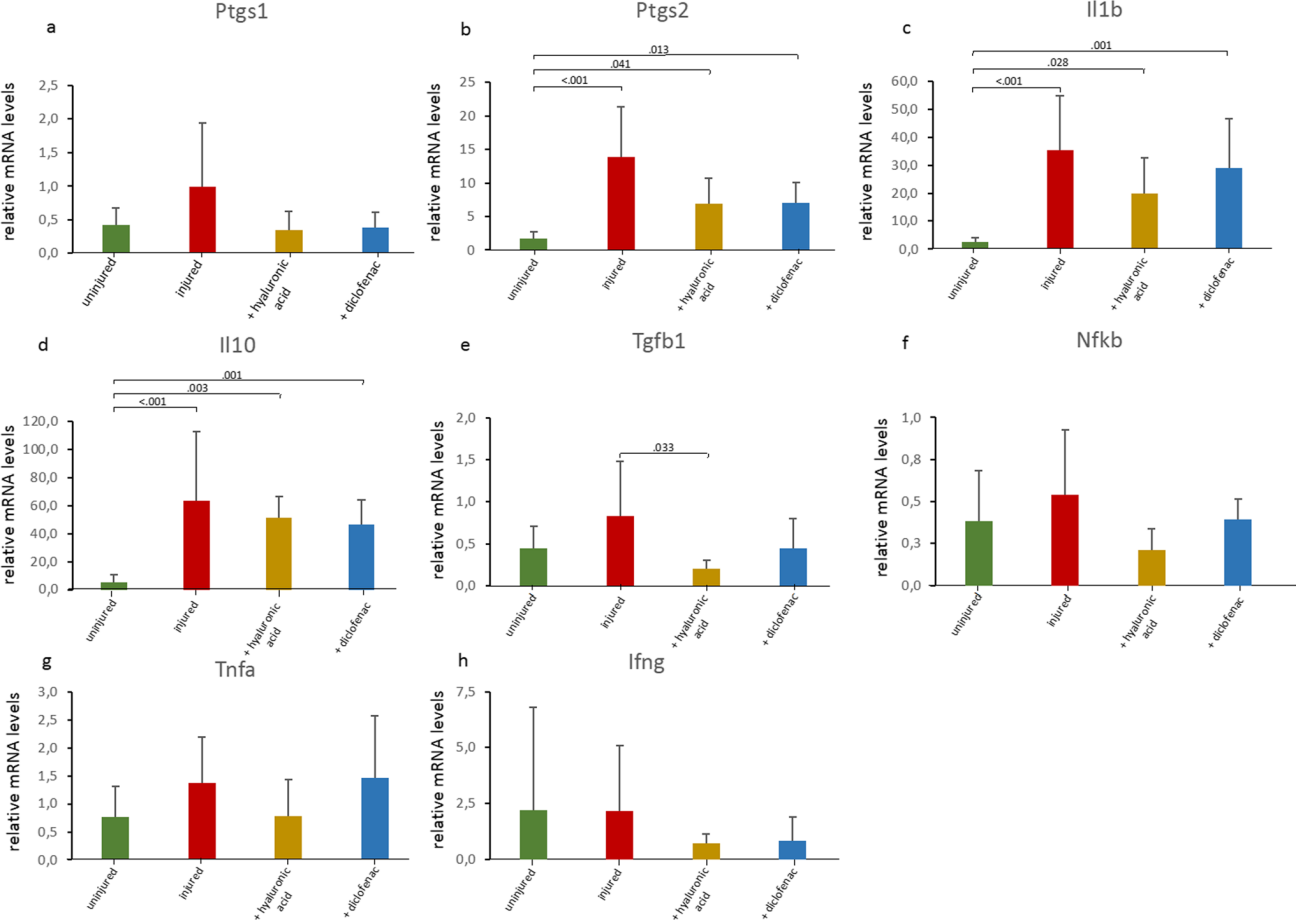
Inflammation-related molecules. Relative gene expression of *Ptgs1* (**a**), *Ptgs2* (**b**), *Tgfb1*(**c**), *Il1b* (**d**), Il10 (**e**), *Nfkb1* (**f**), *Tnfa* (**g**), and *Ifng* (**h**) was measured as fold change compared to the uninjured control group. Results are shown as mean with standard deviation. Kruskal–Wallis test was used for statistical analysis. Where statistically significant, corrected *p* values from post-hoc tests are shown as decimals above the bars. Non-significant *p* values are not shown in this figure, but are reported in the supplementary appendix

The gene expression of cyclooxygenase (*Ptgs*)1 and 2 were upregulated by injury, though only *Ptgs2* reached statistical significance. Both, HA gel and diclofenac gel treatment tended to reduce this upregulation similarly (Fig. 4a, and b, respectively). The gene expression profiles of the interleukins interleukin 1 beta (*Il1b*) and interleukin 10 (*Il10*) were significantly increased after injury, with no statistical effect for either gel treatment (Fig. 4c, and d, respectively). Transforming growth factor beta 1 (*Tgfb1*) gene expression was increased after injury, which was significantly decreased after HA gel treatment and, although not significant, reduced after diclofenac gel treatment (Fig. 4e).

The gene expression profiles of tumor necrosis factor alpha (*Tnfa*), nuclear factor kappa B subunit 1 (*Nfkb1*), and interferon gamma (*Ifng*) remained unchanged after injury and treatment (Fig. 4f–h, respectively).

### ECM-related genes

**Fig. 5:**
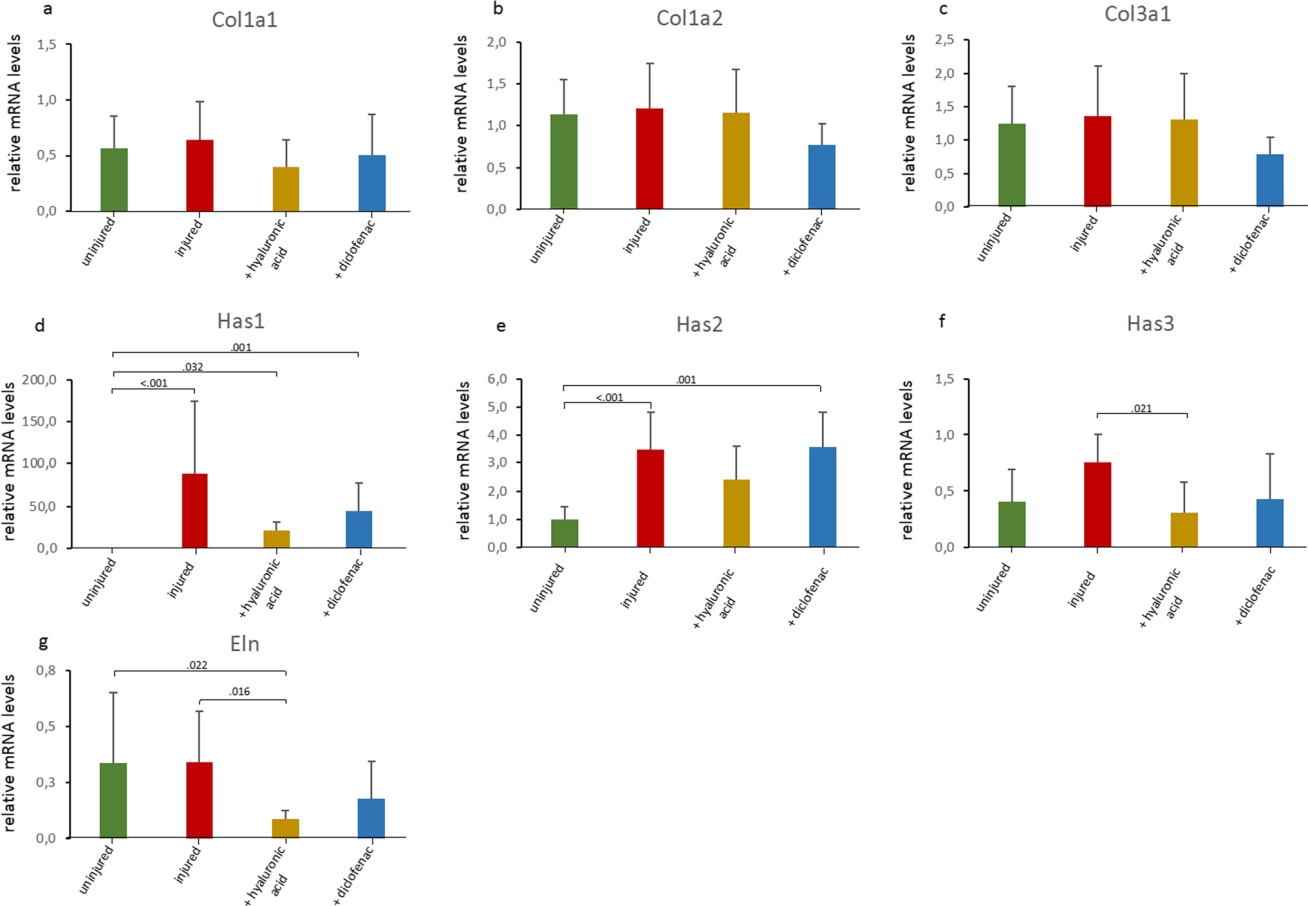
ECM-related molecules. Relative gene expression of *Col1a1* (**a**), *Col1a2* (**b**), *Col3a1* (**c**), *Has1* (**d**), *Has2* (**e**), *Has3* (**f**), and *Eln* (**g**) was measured as fold change compared to the uninjured control group. Results are shown as mean with standard deviation. Kruskal–Wallis test was used for statistical analysis. Where statistically significant, corrected *p* values from post-hoc tests are shown as decimals above the bars. Non-significant *p* values are not shown in this figure, but are reported in the supplementary appendix

The gene expression profiles of collagen type I alpha 1 chain (*Col1a1*), collagen type I alpha 2 (*Col1a2*), collagen type III alpha 1 chain (*Col3a1)* remained unchanged after injury and treatment (Fig. 5a–c, respectively).

Hyaluronan synthase (*Has*) 1 and 2 gene expression levels were significantly upregulated after injury, with no statistical effect for either gel treatment (Fig. 5d, e, respectively). *Has3* gene expression was upregulated after injury without reaching significance, whereas HA gel treatment significantly reduced this upregulation (Fig. 5f).

Elastin (*Eln*) gene expression did not show any change by injury, but was reduced by both gel treatments, whereas the reduction by HA gel treatment reached significance (Fig. 5g).

## Discussion

Significant damage to the VF, including disruption of anatomical structures, can occur in many ways, including (prolonged) intubation or VF surgery. The resulting tissue damage leads to the release of a variety of chemoattractants and factors, such as danger-associated molecular patterns (DAMPs). These play a key role in initiating wound healing. Ultimately, this can lead to loss of physiological organ architecture and tissue fibrosis with the aforementioned reduction in quality of life when the VF is involved [18, 19]. Improving wound healing and ideally preventing fibrosis is, therefore, of paramount importance. The purpose of this study was to evaluate whether a single topical application of HA (with or without diclofenac) immediately after injury has a beneficial effect on postoperative VF wound healing.

### Wound healing

Wound healing can be divided into three partially overlapping phases: inflammatory, proliferative, and tissue remodeling. During the inflammatory phase, immune cells migrate to the site of injury to remove cellular debris and pathogens [3]. In the proliferative phase, fibroblasts produce new ECM to provide structural support for the developing tissue. Finally, during the remodeling phase, excess ECM components are degraded, refining the tissue and forming a mature scar [20, 21].

The foundation of wound healing is laid during the inflammatory phase, as dysregulation can lead to chronic inflammation and impaired wound healing [22]. It is, therefore, particularly attractive to target this phase to improve the functional properties of the remodeled tissue. Indeed, many approaches have been investigated to improve the oscillatory properties of the VF after injury. However, none of these approaches has achieved the desired effect [4, 23]. As a result, there is currently no agent applied in clinical routine in laryngology.

### Inflammatory reaction

The inflammatory phase involves hemostasis and an inflammatory response. Studies have shown that the destruction of keratinocytes leads to the release of IL-1, the body's first signal after tissue injury. This leads to the activation of platelets and the release of other factors, such as growth factors, which are designed to attract more inflammatory cells and thus disarm the infectious agents [19, 24] 

In the present work, a significant upregulation of *Has1*, *Ptgs2*, *Il1b* and *Il10* was seen in all three injury groups compared to the uninjured control group. Similarly, Welham et al. reported a significant upregulation of *Has1*, *Ptgs2*, *Il1b* and *Tnfa* after 1 h of injury [2]. The fact that *Il1b*, *Il10* and *Ptgs2* were significantly upregulated, suggests that the VF injury model in the present study was functional. Although the other inflammatory markers measured (*Ptgs1*, *Tnfa*, *Tgfb1*, *Nfkb*, *Ifng*) are also described as key regulators in the inflammatory phase and would, therefore, be expected to be upregulated, they were not significantly in these experiments [25]. We attribute this to the short observation period.

Tissue repair and remodeling occurs at later stages of wound healing, which explains why there was no significance in the expression of either *COL* or *ELN*. The increase of *Has* expression was, therefore, interesting, as this enzyme is responsible for the production of HA. However, HA is not only a scaffolding protein but plays an important role in immunomodulation and angiogenesis and is, therefore, also relevant at an early stage [26].

### Effects of topical treatment

From a risk–benefit perspective, topical therapy is the most promising, as no systemic side effects are to be expected. Akdogan et al. investigated the effect of topical retinoic acid on VF wound healing in rabbits. They showed that there was a significant reduction in both collagen and fibroblast deposition in the retinoic acid-treated group [18]. However, local irritation has been reported in dermatologic studies, where it is used to treat acne vulgaris [27]. In VF, this side effect can be serious and may lead to significant deterioration of the voice. Therefore, this agent does not appear to be suitable for the treatment of VF scars in humans.

Another study investigated the effect of HA collagen nanofibers on early wound healing. Twelve New Zealand white rabbits underwent unilateral VF injury. The six rabbits in the control group received superficial saline treatment using cotton swabs for three consecutive days, while the other six rabbits were treated with an HA–collagen mixture for three consecutive days. Half of the rabbits in each group (treatment and control) were then sacrificed on day 7, while the other half were euthanized on day 21. Based on a significant reduction in collagen fiber diameter on day 21 in the treatment group, they concluded that topical application of HA–collagen could lead to reduced scar tissue formation [28]. Although an interesting approach, this study may lack statistical power due to the small number of animals in each group. In addition, the sequential application on three consecutive days as described in this publication may not be feasible in clinical routine as the majority of awake patients would probably not tolerate topical treatment and repeated anesthesia for this purpose would simply not be justifiable. Nevertheless, HA appears to be a promising agent for improving VF wound healing and was used in the present study for the reasons described in more detail in the following paragraphs.

HA is a ubiquitous polysaccharide that plays a key role in wound healing and is widely used in other areas of medicine due to its high biocompatibility and low cost [29]. HA is known to inhibit the profibrotic effect of TGFβ1 via the Smad signaling pathway [30]. This may explain the significant downregulation of *Tgfb1* in the present study by the addition of HA to the wound. TGF-β1 plays a critical role in the inflammatory phase of wound healing and excess can lead to hypertrophic scars [31]. Targeting TGF-β1 is, therefore, a popular way to improve wound healing. Studies have shown that inhibition of TGF-β improves wound closure and reduces fibrosis [32, 33]. Therefore, the gene downregulation of *Tgfb1* demonstrated in this study may provide a favorable environment for wound healing.

Our results showed that *Eln*, which is also partially regulated by Smad [34], was significantly downregulated by the addition of HA. ELN is urgently needed for tissue repair, but it appears that the composition of the cross-links is more important than the concentrations themselves. [35] Based on the available results, it is not possible to make a conclusive assessment in this regard and further investigation is required.

In the presence of HA, *Has* was downregulated, but only *Has3* was significant. This may suggest a negative feedback mechanism to maintain HA homeostasis. According to Tammi et al. the relationship between HA concentration and *Has* expression is poorly understood. This may be due to technical difficulties in reliably detecting HAS at the protein level [36]. Therefore, this feedback mechanism can currently not be demonstrated in a healthy population.

In the group, where a mixture of HA and diclofenac was applied to the wound, no significant changes were observed compared to the injury group. Diclofenac belongs to the group of NSAIDs and exerts its anti-inflammatory effect through COX inhibition. However, the effect of topical diclofenac on wound healing is controversial. While the anti-inflammatory effect may be beneficial, histomorphometric studies have shown a reduction in fibroblasts, which may be associated with impaired wound healing [37, 38]. The lack of significance with the addition of diclofenac in the present study could have several reasons. These include the short observation period, the low dose, or an actual lack of effect.

### Limitations

There are some limitations to this study. One is that the observation period was very short. To study the effect of the drugs over a longer period of time, the animals would have had to be awakened from anesthesia. This was impossible, because the gel would have blocked their airways and they would have suffocated. Therefore, it is not possible to draw definitive conclusions about the effects of the local therapy used here on the fibrotic processes and thus on the vibrational properties of the VF. One way to overcome these problems in the future would be to use larger animals. Rats were used in this work, because they have a very similar structure to the human VF [39]. At the cost of greater histological differences, other animal models (e.g., sheep) could more easily be used to study long-term effects. Another limitation is that only changes at the mRNA level were examined. This was also due to the small size of the rat larynx, as only very little tissue could be obtained. This could have been achieved by increasing the number of animals or switching to another animal model. The latter would be preferable, because it would require fewer animals, taking into account the principle of 3Rs (reduce, replace, refine).

## Conclusion

Topical therapy is a promising option in a risk–benefit analysis to prevent postoperative scarring after VF surgery. The immediate and single postoperative application of a substance may not only be easy to implement for the surgeon, but more importantly, may be best for the patient. Similar to studies in other medical fields, the results suggest that the postoperative use of HA may have a beneficial effect on wound healing [29, 40, 41]. However, further studies with longer observation periods including histological examinations are needed to make a definitive assessment.

### Supplementary Information

Below is the link to the electronic supplementary material.

Supplementary file1 (XLSX 120 KB)

## Data Availability

Not applicable.
